# Analysis of Cesarean Section Rates in a Public Tertiary Hospital During Teaching and Non-teaching Periods Using the Robson Ten Group Classification System

**DOI:** 10.7759/cureus.43838

**Published:** 2023-08-21

**Authors:** Sepideh Rezaei Ghamsari, Elham Taeidi, Fatemeh Darsareh, Vahid Mehrnoush

**Affiliations:** 1 Department of Midwifery and Reproductive Health, Tehran University of Medical Sciences, Tehran, IRN; 2 Mother and Child Welfare Research Center, Hormozgan University of Medical Sciences, Bandar Abbas, IRN

**Keywords:** non-teaching hospitals, teaching hospitals, cesarean indications, cesarean section, robson

## Abstract

Introduction: The rising cesarean section (CS) rate is a global concern. One of the hospital characteristics that may explain the variation in CS among hospitals is hospital teaching status. This study aims to assess the rate of CS in a tertiary hospital during the teaching and non-teaching periods and to conduct an analysis using the Robson ten-group classification system.

Methods: This study is a retrospective cohort that assessed pregnant mothers who gave birth at a tertiary hospital in Bandar Abbas. The study population was divided into two groups: those who gave birth during the hospital's teaching period (November 1st, 2019 to October 30th, 2020) and those who gave birth after that (November 1st, 2020 to October 30th, 2021). The primary outcome was the rate of CS according to Robson's classification system. The secondary outcome was the contributions of each group of Robson to the overall CS rate. Data were extracted by trained collectors from the "Iranian Maternal and Neonatal Network (IMaN Net)," a valid national system, using electronic patient records.

Results: Of the total number of births (8382), 62.9 % occurred during the teaching period and 37.1 % during the non-teaching period. A 7% increase in CS was observed during the teaching period of the hospital compared to the non-teaching period (p<0.01). CS rate in Robson groups 1,2,4,7, and 10 differs significantly between teaching and non-teaching periods. According to the findings, Groups 5, 10, and 2 were the three most significant contributors to overall CS in our hospital during the study period.

Conclusion: The efforts to reduce the overall CS rate should be focused on groups 2,5, and 10 of Robson.

## Introduction

Cesarean section (CS) is a critical intervention for improving health outcomes of high-risk pregnancies and lowering maternal and neonatal morbidity and mortality. Therefore, it is one of the most accurate predictors of the quality of maternal health care [1]. On the other hand, the rising CS rate is a global concern. In the last two decades, population CS rates varied considerably across regions, ranging from dangerously low rates, such as 4.1% in West and Central Africa, to extraordinarily high rates, such as 44.3% in Latin America [2]. New data on first-time pregnant women, term, singleton and cephalic presentation CS in Iran show a high tendency among health authorities, with significant and consistent intra-regional variation [3,4]. One of the hospital characteristics that may explain the variation in CS among hospitals is hospital teaching status. Teaching hospitals have higher overall rates of surgery, which may be due to more complex caseloads [5]. As a result, it is reasonable to assume that teaching hospitals have a higher proportion of high-risk births and, as a result, a higher likelihood of CS [6].

Khaleej-e-Fars hospital in Bandar Abbas, Iran, has had teaching and non-teaching experiences. Due to internal provincial policies, it merged with the province's only reference training hospital from June 2016 to November 2020. This merge resulted in significant changes in the hospital's birth rates, causing the CS index to fluctuate significantly. We hypothesized that becoming a teaching hospital and having obstetric and gynecological residents manage a large portion of patient care, and treatment significantly impacted changing the CS index. As a result, we decided to investigate the indications for CS using the Robson system. This study aimed to assess the rate of CS in a tertiary hospital in Bandar Abbas, Iran, during the teaching and non-teaching periods and to conduct an analysis using the Robson ten-group classification system.

The Robson classification system (also known as the 10-point scale) divides all deliveries into ten mutually exclusive and completely inclusive groups based on predefined obstetric parameters. Parity, previous CS, the onset of labor, fetal presentation, number of fetuses, and gestational age are all factors to consider [7,8].

Since 2001 when the Robson classification was proposed, many facilities and countries have adopted it to monitor CS rates in their populations and evaluate the impact of management changes that may affect these rates [7]. Recognizing its benefits and simplicity, the Robson classification system was recommended as a global standard for analyzing the trends in the overall and group-specific CS rates over time by the World Health Organization (WHO) and the International Federation of Gynecology and Obstetrics (FIGO) [8,9].

## Materials and methods

A cross-sectional study was carried out at Khaleej-e-Fars Hospital, a public tertiary care facility that handles about 4000 deliveries per year, in Bandar Abbas, Iran. Our study population was divided into two groups: those who gave birth during the hospital's teaching period (November 1st, 2019 to October 30th, 2020) and those who gave birth after that (November 1st, 2020 to October 30th, 2021). The primary goal was to find out how common CS was in each group using Robson's classification. The secondary objective was to see if there were any differences in the contributions of each group to the overall CS rate between teaching and non-teaching periods of hospitalization.

The Robson classification system (also known as the 10-point scale) divides all deliveries into 10 mutually exclusive and completely inclusive groups based on predefined obstetric parameters. Parity, previous CS, the onset of labor, fetal presentation, number of fetuses, and gestational age are all factors to consider (Table 1).

**Table 1:** Robson Classification System CS: Cesarean section

Group	Obstetric population
1	Nulliparous women with a single cephalic pregnancy, ≥37 weeks gestation in spontaneous labor
2	Nulliparous women with a single cephalic pregnancy, ≥37 weeks gestation who had labor induced or were delivered by CS before labor
3	Multiparous women without a previous CS, with single cephalic pregnancy, ≥37 weeks gestation in spontaneous labor
4	Multiparous women without a previous CS, with a single cephalic pregnancy, ≥37 weeks gestation who had labor induced or were delivered by CS before labor
5	All multiparous women with at least one previous CS, with a single cephalic pregnancy, ≥37 weeks gestation
6	All nulliparous women with a single breech pregnancy
7	All multiparous women with a single breech pregnancy, including women with previous CS (s)
8	All women with multiple pregnancies, including women with previous CS (s)
9	All women with a single pregnancy with a transverse or oblique lie, including women with previous CS (s)
10	All women with a single cephalic pregnancy < 37 weeks gestation, including women with previous CS (s)

Data were extracted by trained collectors from the "Iranian Maternal and Neonatal Network (IMaN Net)," a valid national system, using electronic patient records. First, the institution's overall CS rate was calculated. All data collected was coded, and women were assigned to one of the 10 Robson groups. The group's size concerning the total obstetric population, contribution to the overall CS rate, and CS rate within the group were all calculated. Information on socio-demographic factors (age, education, living place, and health insurance) and obstetrical factors (parity, use of assisted reproductive technology, high-risk pregnancy) were extracted from electronic patient records. Those with a history of diabetes, hypertension, preeclampsia, cardiovascular disease, severe anemia, a BMI greater than 30 kg/m^2^, drug addiction, chorioamnionitis, or coronavirus disease 2019 COVID-19 were considered high risk.

SPSS Statistics for Windows, version 19.0 (IBM Corp., Armonk, NY) was used to export and analyze data (Illinois, USA). Descriptive statistics were presented as frequencies and proportions. The number of births and proportion of CS within each group of Robson was given and further stratified. To assess differences in proportions of CS by the hospital's teaching and non-teaching period, the χ2 tests within each Robson group were performed.

## Results

Of the total number of births (8382), 62.9 % occurred during the teaching period and 37.1 % during the non-teaching period. Table 2 shows the socio-demographic characteristics of the study population, which were not significantly different.

**Table 2:** Socio-demographic characteristics of study populations

Variables	All	Teaching period	Non-teaching period	P-value
Age (Year) (mean ± SD)	29.63±3.93	30.17±4.5	28.73±2.98	0.139
Age range	n (%)	n (%)	n (%)	0.171
Less than 18	945 (11.3%)	547 (10.4%)	398 (12.8%)	
18-35	6149 (73.3%)	3976 (75.4%)	2173 (69.9%)	
More than 35	1288 (15.4%)	750 (14.2%)	538 (17.3%)	
Education	n (%)	n (%)	n (%)	0.098
Illiterate	291 (3.5%)	117 (2.2%)	174 (5.6%)	
Primary	819 (9.7%)	542 (10.3%)	277 (8.9%)	
High school/Diploma	4592 (54.8%)	3066 (58.2%)	1526 (49.1%)	
Advanced	2680 (32%)	1548 (29.3%)	1132 (36.4%)	
Health Insurance	n (%)	n (%)	n (%)	0.861
Yes	7632 (91.1%)	4809 (91.2%)	2823 (90.8%)	
No	750 (8.9%)	464 (8.8%)	286 (9.2%)	
Living place	n (%)	n (%)	n (%)	0.053
Urban	5157 (61.5%)	2984 (56.6%)	2173 (69.9%)	
Rural	3225 (38.5%)	2289 (43.4%)	936 (30.1%)	

Table 3 depicts the obstetrical characteristics of the study population. It was discovered that those who gave birth during the teaching period did not differ statistically in terms of parity or use of assisted reproductive technology. However, more high-risk pregnant women were admitted to the hospital during the teaching period (P<0.001).

**Table 3:** Obstetric characteristics of study populations

Obstetrical characteristics	All	Teaching period	Non-teaching period	P-value
Parity	%	%	%	0.108
Nulliparous	25.1%	24.6%	26%	
Multiparous	74.9%	75.4%	74%	
Assisted-reproductive Technology	%	%	%	0.741
Yes	4.7%	5.1%	4%	
No	95.3%	94.9%	96%	
High-risk Pregnancy	%	%	%	0.001
Yes	33.1%	40.3%	20.9%	
No	66.9%	59.7%	79.1%	

Table 4 presents the birth trend of the hospital during the study period. A total of 4337 CS were performed, yielding an overall CS rate of 42.8%, ranging from 45.4% during the teaching period to 38.4% during the non-teaching period. Based on χ2 tests, the rate of CS was significantly different between the teaching and non-teaching period (p<0.01). According to the Robson ten group classification, groups 3,5,1, and 2 were the most populous, accounting for 26.6%, 19.8%, 12.7%, and 12.4% of the total study population. Table 4 also shows the number of CS in each Robson classification system group in the study population. As we can see, the CS rate in Robson groups 1,2,4,7, and 10 differs significantly between teaching and non-teaching periods.

**Table 4:** Number of CS in each group of the Robson classification system in the study population * The total number of birth in each group of Robson. ** Number of cesarean sections in each group of Robson. CS: Cesarean section

Robson Classification System	All			Teaching			Non-teaching			P-value
	Birth (n)*	CS (n)**	%	Birth (n)*	CS (n)**	%	Birth (n)*	CS (n)**	%	
Group 1	1063	257	22.6	664	192	28.9	399	65	16.3	0.001
Group 2	1042	445	40.6	632	296	46.8	410	149	36.3	0.001
Group 3	2231	154	6.4	1385	119	8.6	846	35	4.1	0.009
Group 4	945	146	14.5	578	109	18.9	367	37	10.1	0.012
Group 5	1661	1654	99.5	1007	1004	99.7	654	650	99.4	0.253
Group 6	124	105	85.1	79	66	83.5	45	39	86.7	0.09
Group 7	141	130	89.6	103	98	95.1	38	32	84.2	0.001
Group 8	168	153	91.9	117	105	89.7	51	48	94.1	0.051
Group 9	26	26	100	15	15	100	11	11	100	NA
Group 10	981	515	50.4	693	388	56	288	127	44.1	0.001
Total	8382	3585	42.8	5273	2392	45.4	3109	1193	38.4	0.003

The contribution of each group in the Robson classification system to the overall CS prevalence in the study populations is shown in Table 5. Women in group 5 were the most significant contributor to the overall CS rates (46.1%), ranging from 44% in the teaching period to 54.5% in the non-teaching period. The second most substantial contributors were women in group 10 (14.4%), ranging from 16.2% in the teaching period to 10.6% in the non-teaching period. The third-largest group was group 2, representing 12.4% of the study population, ranging from 12.4% in the teaching period to 12.5% in the non-teaching period.

**Table 5:** Contribution of each group in the Robson classification system to the overall CS prevalence in the study populations CS: Cesarean section

Robson Classification System	All (%)	Teaching (%)	Non-teaching (%)
Group 1	7.2	8	5.5
Group 2	12.4	12.4	12.5
Group 3	4.3	5	2.9
Group 4	4.1	4.5	3.1
Group 5	46.1	42	54.5
Group 6	2.9	2.8	3.3
Group 7	3.6	4.1	2.7
Group 8	4.3	4.4	4
Group 9	0.7	0.6	0.9
Group 10	14.4	16.2	10.6

## Discussion

According to the Robson classification system, the CS rate among nulliparous women in Groups 1 and 2 during teaching and non-teaching periods was significantly higher than the WHO recommendation. It has been recommended that the CS rate in nulliparous women with a single cephalic pregnancy at 37 weeks of gestation or more in spontaneous labor be less than 10% (1), but our hospital had a rate of 22.6%. When we compared the teaching and non-teaching periods, we found that the rate was higher during the teaching period (28.9% vs 16.3%). The same findings were discovered in nulliparous women with a single cephalic pregnancy, at 37 weeks of gestation or more, who had labor induced or were delivered by CS before labor. The large number of primary CS in Groups 1 and 2 becomes even more significant to the future overall CS rate. As a result, efforts to halt the rising trend of CS rates must address these groups to be successful [10]. The detailed indications of CS among primiparous women (Groups 1 and 2) may aid in our understanding of the situation. This is especially important because women in these groups are considered to be low-risk.

The rate of CS among multiparous women without a previous CS, with a single cephalic pregnancy, ≥37 weeks of gestation in spontaneous labor (Group 3) were above the recommended rate (less than 3%) (1), with no differences between teaching and non-teaching period. The rate of CS in multiparous women without a previous CS, with a single cephalic pregnancy, ≥37 weeks of gestation who had labor induced or were delivered by CS before labor (Group 4) should not exceed 15%; according to WHO recommendations (1), however, this rate was 18% during teaching period of our hospital. Some speculated that the higher CS rates in this group were due to insufficient data collection. It is possible that women with previous scars (Group 5) were mistakenly assigned to Group 3 or 4 [11]. Other possible explanations for high rates include maternal requests [3]. 

Rates of 50-60% are considered appropriate for all multiparous women with at least one previous CS, a single cephalic pregnancy, and ≥37 weeks of gestation (Group 5) (1). In our hospital, this rate was 99.5%. This could be because most obstetricians at our hospital have a policy of scheduling pre-labor CS for all women with one previous scar without attempting a trial of labor. In countries with a higher rate of vaginal birth after cesarean (VBAC), the rate of CS in Group 5 is less [11].

In our hospital, approximately 85% of nulliparous women with a single breach pregnancy (Group 6) were delivered by CS, with no statistically significant difference between the teaching and non-teaching periods. The same results were observed in Group 7. The CS rate among Group 8 (women with multiple pregnancies, including women with previous CS) was higher than the recommended rate, which is around 60% (1), with no differences between teaching and non-teaching period (89% vs 94.1%). This high rate is due to our obstetricians' policy of scheduling all multiple pregnancies for CS without a labor trial. All women with a single pregnancy with a transverse or oblique lie, including women with previous CS (Group 9), had CS.

It has been suggested that approximately 30% of women with a single cephalic pregnancy, less than 37 weeks of gestation, including women with previous CS (s), would be subjected to CS (1). In our hospital, the rate was higher than expected, with a greater tendency during the teaching period compared to the non-teaching period (56% vs 44.1%).

According to the findings, Groups 5, 10, and 2 were the three most significant contributors to overall CS in our hospital during the study period. Understanding how CS rates vary by hospital characteristics may aid in understanding and possibly modifying some structural and process components of childbirth services to reduce the need for CS. One of the hospital characteristics that may explain the variation in CS among hospitals is hospital teaching status. There have been studies on the relationship between hospital teaching status and CS. In 1986, the first report on a link between a hospital's teaching status and the likelihood of a CS was published. Women delivering in hospitals with teaching status were less likely to have a primary CS than women giving birth in hospitals without this designation, even after controlling for the mother's age at childbirth, the presence of pregnancy, labor, and childbirth complications, the expected primary payer, and the size of the hospital. Women of all age groups had a significantly lower CS rate in teaching hospitals [12]. Another survey conducted to investigate the CS rate in teaching hospitals in the United States and the factors that may influence it discovered that the estimated CS in all hospitals with obstetrics and gynecology residencies was 20.3%, compared to a national rate of 23.5%. Women who gave birth in teaching hospitals were less likely to have a CS than women who gave birth in hospitals that did not have residency programs [13]. There was also a difference in the proportion of CS between teaching and non-teaching hospitals [14]. According to a recent meta-analysis, teaching hospitals perform fewer CS in various countries for various study populations and population characteristics [8]. In general, teaching hospitals are associated with a higher standard of care and better outcomes [12,15]. Evidence-based guidelines and clinical protocols are being followed more closely in teaching hospitals. Teaching hospitals also generally have access to a certain level of technology, making it easier to implement safeguards requiring a second opinion on CS procedures. As a result of fewer unnecessary procedures, higher quality care can also translate to a lower likelihood of CS [12].

However, our findings contradicted previous research. A 7% increase in CS was observed during the teaching period of the hospital compared to the non-teaching period. Two main factors could explain this. First, as the province's only referral hospital, all patients, including those with high-risk pregnancies, were admitted to the hospital during the teaching period. Teaching hospitals are known to have higher overall rates of surgery, which may be due to more complex caseloads [16]. As a result, it is reasonable to assume that teaching hospitals have a higher proportion of high-risk births and, as a result, a higher likelihood of CS. Second, delegating a large portion of patient care to obstetricians may increase cesarean section rates. Residents' lack of experience managing high-risk pregnancies and performing complex deliveries, fear of instrumental deliveries, and strong desire for a cesarean section, particularly in the early years of residency, can all contribute to this. The high c-section rate in Group 5 reveals that this hospital followed the motto "once a c-section, always a c-section," passing up the opportunity to teach Vaginal Birth After Cesarean (VBAC) and possibly prioritizing teaching surgical techniques. Adopting more VBAC trials by obstetricians may help to reduce the CS rate not only in Robson group 5 but also in the overall rate of CS over time. Furthermore, effective labor induction management, selecting vaginal delivery for appropriate breech presentations and multifetal pregnancies, proper obstetrician education for operative vaginal delivery, and objective evaluation of labor dystocia may be key points in reducing the CS rate [17]. Further investigation may assist us in better understanding the reasons.

The strength of our study is that we took into account the main demographic confounders to compare the differences between teaching and non-teaching hospital status. In addition, other maternal and fetal factors that significantly influence the rate of CS (e.g. maternal age, pre-existing conditions such as BMI, or complications) are considered. The primary Robson classification identifies the contributors to the CS rate but does not provide insight into the reasons (indications) or explanations for the differences observed [1,8]; thus, additional statistical methods (e.g. adjusting) are required to account for these factors which are the limitation of our study. Our findings should be preliminary, and our hypothesis should be tested in other populations and states. However, this study shows a need for a more specific understanding of hospital teaching status's programmatic, technological, and staffing factors that explain its association with a higher risk of CS.

## Conclusions

According to the findings, Groups 5, 10, and 2 were the three most significant contributors to overall CS in our hospital during the study period. The efforts to reduce the overall cesarean section rate should be focused on these groups. Our findings should be preliminary, and our hypothesis should be tested in other populations and other hospitals with different characteristics. However, this study shows a need for a more specific understanding of hospital teaching status's programmatic, technological, and staffing factors that explain its association with a higher risk of CS.
